# Graded Microstructure and Mechanical Performance of Ti/N-Implanted M50 Steel with Polyenergy

**DOI:** 10.3390/ma10101204

**Published:** 2017-10-19

**Authors:** Jin Jie, Tianmin Shao

**Affiliations:** State Key Laboratory of Tribology, Tsinghua University, Beijing 100084, China; jjking200346@163.com

**Keywords:** ion implantation, M50 bearing steel, friction and wear, gradient structure materials, nano-hardness

## Abstract

M50 bearing steels were alternately implanted with Ti^+^ and N^+^ ions using solid and gas ion sources of implantation system, respectively. N-implantation was carried out at an energy of about 80 keV and a fluence of 2 × 10^17^ ions/cm^2^, and Ti-implantation at an energy of about 40–90 keV and a fluence of 2 × 10^17^ ions/cm^2^. The microstructures of modification layers were analyzed by grazing-incidence X-ray diffraction, auger electron spectroscopy, X-ray photoelectron spectroscopy, and transmission electron microscopy. The results showed that the gradient structure was formed under the M50 bearing steel subsurface, along the ion implantation influence zone composed of amorphous, nanocrystalline, and gradient-refinement phases. A layer of precipitation compounds like TiN is formed. In addition, nano-indentation hardness and tribological properties of the gradient structure subsurface were examined using a nano-indenter and a friction and wear tester. The nano-indentation hardness of N + Ti-co-implanted sample is above 12 GPa, ~1.3 times than that of pristine samples. The friction coefficient is smaller than 0.2, which is 22.2% of that of pristine samples. The synergism between precipitation-phase strengthening and gradient microstructure is the main mechanism for improving the mechanical properties of M50 materials.

## 1. Introduction

The bearings of aero engines operate in alternating high-speed and high-contact stress environments, in which damage could arise from contact fatigue, wear, and corrosion [1]. M50 is one of the main bearing steels currently used in aero engines due to its higher impact resistance, contact fatigue strength, and high-temperature-bearing performance. However, wear remains one of the main failure modes of the bearing materials [2]. Surface modification can efficiently improve the surface mechanical properties of bearing materials, in terms of reducing the friction coefficient and improving wear resistance. In recent years, new surface modification technologies have been developed for application to bearings in order to improve the performance of main shaft bearings in aero engines operating under harsh environments [3,4,5,6].

Ion implantation is one of the most suitable technologies for surface modification of bearing components, improving their wear-resistance [7], surface hardness [7,8], and contact fatigue [8] due to its unique advantage of maintaining the size of the workpiece and working accuracy during ion implantation [9]. Generally, the range of energy used for ion implantation ranges from 20 keV to 100 keV [10,11,12,13], and the mechanism of enhancing wear resistance of metal materials by ion implantation is believed to be associated with the increase of surface hardness and decrease of friction coefficient. On the other hand, the improvement in fatigue failure resistance is related to surface hardness, strength, dislocation locking, solution strengthening, and irradiation damage [14]. In the early stages, many scholars focused on macroscopic properties like friction and wear [15,16,17]. However, research on the microscopic mechanism of wear resistance in the implanted layer is still lacking, and existing viewpoints are still controversial. Xie et al. proposed a theory about the migration of elements that suggests that implantation produces more vacancy defects. At higher contact stresses, the elements migrate along the vacancy defects, thereby improving the wear resistance of the modified layer [18]. Sharkeev et al. suggested that ion implantation has long-range effects, including ion bombardment forming dense dislocations, vacancy defects, and grain refinement in areas much thicker than the depth of element distribution. It significantly improves the strength and wear resistance of the material [19]. Some researchers have also pointed out that it is difficult to improve the performance of the implanted layer by migration alone. The long-range effect of ion implantation dramatically improves the performance of the ion-implanted layer [20].

The shallower subsurface layer formed by ion implantation is unfavorable for surface strengthening. Furthermore, the different microstructures have a key role in the performance of the surface-modified layer of the bearing. An implantation-induced single structure has limited capacity to improve the performance of bearing materials. The mechanical properties of metals can be greatly improved using nano-gradient structure materials [21,22,23]. However, there has been little research conducted on the mechanism for the improvement in the mechanical properties of the gradient microstructure of the implanted layer. In addition, there are few reports about whether gradient microstructures with the pull tail phenomenon can improve mechanical properties [6,7]. In particular, few studies on ion implantation with polyenergy in M50 bearing steels have yet been reported. Large improvements in the mechanical properties for Zr/N-implanted M50NiL have been reported [8]. Thus, the effective parameters in [8] were applied to our study for the expected improvement for low-carbon steel M50 in this study.

However, as reported [7], the Ti ions penetrated more deeply than Zr for single-element-implantation. Therefore, in this study, we have used the polyenergy implantation of N^+^ and Ti^+^ ions to treat M50 bearing materials. Moreover, this study will elucidate the strengthening effect of gradient microstructures, which was barely investigated in [8]. Thus, the structure and composition along the depth of the implanted layer were investigated. The mechanical properties were evaluated using nanoindentation, friction, and wear tests. Finally, the microstructure and strengthening mechanism of the polyenergy ion implantation layer were investigated in detail.

## 2. Materials and Methods

### 2.1. Preparation of Samples

M50 steel is prepared by double vacuum smelting and vacuum heat treatment. Its chemical composition is shown in Table 1. The samples were pretreated as follows: The substrates were cut into Φ 20 mm × 8 mm plates before ion implantation, and the specimens were polished with 600#, 800#, 1200#, 1500#, and 2000# sandpaper and diamond abrasive paste with particle sizes of 0.5–2.5 μm. The final value of roughness of substrates (*Ra*) is about 3 nm. Finally, after ultrasonic cleaning for 10 min with analytical-grade pure acetone and alcohol, the samples are dried in a vacuum furnace.

**Figure 1:**
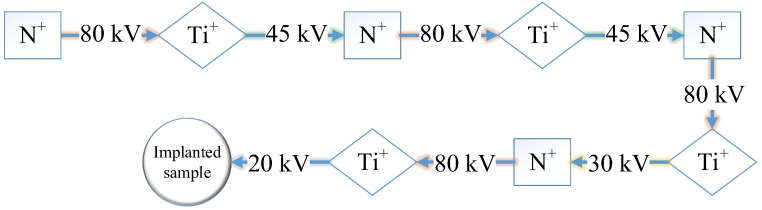
The fabricating process of N^+^ + Ti^+^ ion implantation for M50 steel.

As shown in Figure 1, polyenergy mode is adopted during the N^+^ and Ti^+^ co-implantation. Ti^+^ ion is implanted using a metal vapor vacuum arc (MEVVA) ion source, while N^+^ ion is implanted by an electron cyclotron resonance (ECR) gas ion source. The implantation parameters are shown in Table 2. In this study, the mean valence state of N and Ti is about +1 and +2, respectively, as per the time-of-flight method. Thus, the energy of N ions is about 80 keV at accelerating voltage of 80 kV and that of Ti ions about 40–90 keV at accelerating voltage of 20–45 kV.

### 2.2. Characterization Methods

The friction and wear performances were investigated using a ball-on-disc tribometer (MTS-3000, Lanzhou Institute of Chemical Physics, Chinese Academy of Sciences, Lanzhou, China). The experimental parameters were as follows: The counterpart was an M50 steel ball. The applied normal load and sliding speed were 0.5 N and 100 r/min, respectively, with the rotation radius of 4.5 mm, at room temperature (25 °C) with a relative humidity of 30%. A nano-indentation (Agilent G200, Agilent Technologies Inc., Santa Clara, CA, USA) was used to evaluate the surface nano-hardness of the pristine and implanted samples. The indentation velocity of the indenter and the transverse strain velocity were 10 nm/s and 0.05 s^−1^, respectively. The harmonic displacement amplitude was set at 2 nm, and the frequency was 45 Hz.

D8 ADVANCE X-ray diffraction (XRD) (Bruker, Karlsruhe, Germany) was used to detect the crystallite structure after implantation. The Cu target monochromatic X ray source (Cu Kα = 1486.4 eV) was used for normal and grazing-incidence XRD analyses. The grazing-incidence angle was 1°.

XPS (PHI Quantera SXM) (ULVAC-PHI Inc., Chigasaki, Japan) with an Al target was used to analyze the chemical compositions of implanted samples. The etching rate was 15 nm/min with an accelerating voltage of 4 kV, which was calibrated using SiO_2_ film. The focused X-ray beam size was 100 μm and the associated energy was 55 eV. In addition, the vacuum pressure in the analysis chamber was higher than 1.33 × 10^−5^ Pa.

The concentration distributions of the elements along the implantation depth were detected using AES (PHI-700) (ULVAC-PHI Inc., Chigasaki, Japan). The accelerating voltage of the electron gun was 5 kV, and the energy resolution was 1‰. The vacuum pressure in the analysis chamber was higher than 5.19 × 10^−7^ Pa. An Ar sputter gun was used to etch the sample surface with a sputtering rate of ~13 nm/min, which was calibrated by referencing to SiO_2_/Si film.

To elucidate the structure of implanted zone at different depths, the cross sections of both samples were examined using HRTEM technique on JEOL JEM 2010F equipment (Japan Electron Optics Laboratory (JEOL), Tokyo, Japan). The pretested cross-sectional samples were prepared using focused ion beam technique (TESCAN LYRA 3 FEG-SEM/FIB) (TESCAN Inc., Brno, Czech Republic). The samples were trimmed to the size of 10 μm × 5 μm by a gallium-ion sputtering gun with beam energy of 30~50 keV, followed by fine milling to obtain samples thin enough for HRTEM test with a series of lower-energy sputtering. To protect the surface from oxidation in the test, a thin SiO_2_ film was deposited.

## 3. Results and Discussion

### 3.1. Mechanical Performance

**Figure 2:**
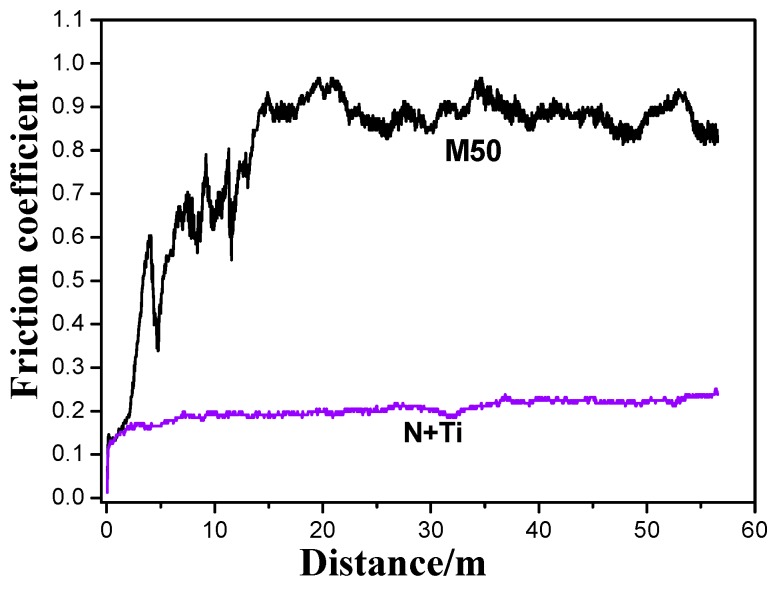
Comparison of the friction coefficient curves before and after N^+^ + Ti^+^ ion implantation for M50 steel.

Figure 2 shows the friction coefficient curves of the samples with and without implantation. These indicate that the friction coefficient of the pristine M50 sample initially increased rapidly to around 0.9, mainly due to the loss of the surface oxide layer and the adsorption layer. Then, the friction coefficient remained stable during the rest of the experiment. It reveals the severe rubbing process and intensive interfacial interactions between the M50 plate and the M50 steel ball. In comparison, the friction process was quite stable for the sample implanted with N^+^ and Ti^+^ ions, with a friction coefficient of 0.1–0.2 maintained during the entire distance of 56.5 m, indicating a good friction reduction capacity and wear.

**Figure 3:**
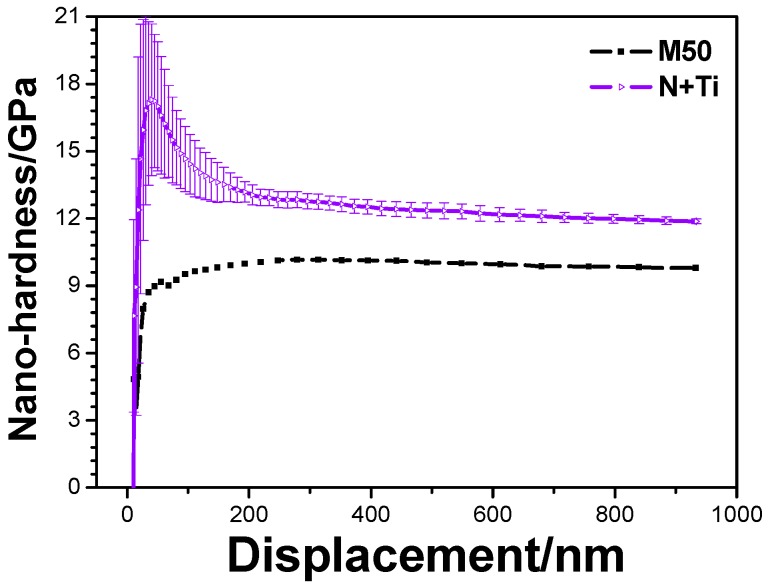
Nanoindentation curves of the samples before and after N^+^ + Ti^+^ ion implantation.

Results of nanoindentation tests are shown in Figure 3. The results show that the nano-hardness of the pristine M50 sample was ~9 GPa. After the polyenergy implantation of N^+^ + Ti^+^, the maximum hardness of 17.8 GPa was achieved for the subsurface layer (thickness of ~30 nm). Subsequently, the hardness decreased with increasing indentation depth. When the indentation depth was in the range of 200–900 nm, the nano-hardness could be stabilized at 12.0 GPa, which was ~3 GPa more than that of the pristine M50. The results of this study are quite consistent with those reported in the literature [15].

### 3.2. Phase Constitution of Surface Layer

**Figure 4:**
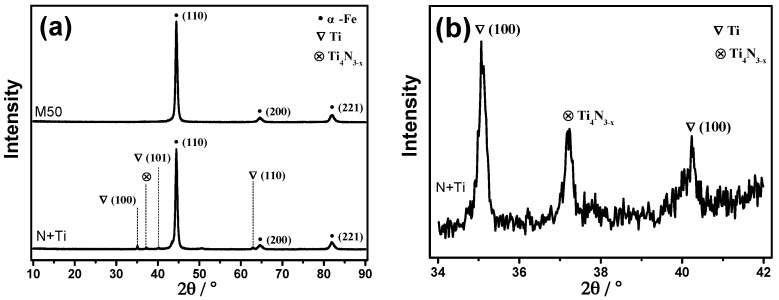
XRD spectra of pre- and post-implantation M50. (**a**) Normal scan; (**b**) Fine scan in the range of 34°–42°.

The XRD data of the substrate and implanted samples are shown in Figure 4. Figure 4a shows three obvious peaks at the positions of 44.5°, 66.2°, and 81.6°. Since the M50 material is mainly composed of martensite after heat treatment, the peaks may correspond to body centred cubic (BCC) α-Fe(110), α-Fe(200), and α-Fe(211) [20], respectively. In addition, a fine scan in the 34–42° range (Figure 3b) revealed that the peak attributed to the Ti_4_N_3-x_ phase appears at 37.3°, and that metallic Ti exists simultaneously. This is similar to the presence of metallic Ti in 9Cr18Mo samples with conventional N^+^ + Ti^+^ implantation [23]. Thus, it can be attributed to the substitution of Fe by Ti, besides the formation of the Ti_4_N_3-x_ ceramic phase.

**Figure 5:**
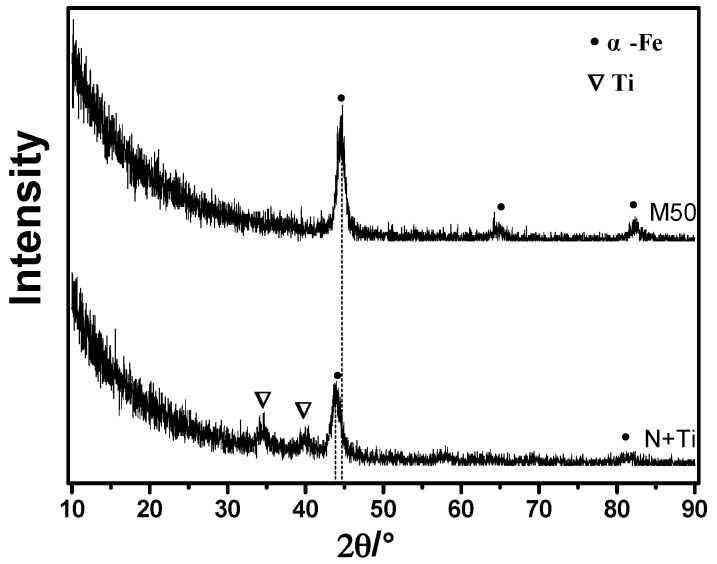
Grazing-incidence XRD results of pristine M50 and implanted sample with an incidence angle of 1°.

To further elucidate the phase structure in the near-surface region, a small-angle grazing-incidence XRD (GXRD) analysis was performed, and the results are shown in Figure 5. The GXRD results reveal the structures of the implantation-affected layer. It was found that metallic Ti phase was formed in the implantation layer, with characteristic peaks at 34.6°and 39.8°.

### 3.3. Chemical Composition

**Figure 6:**
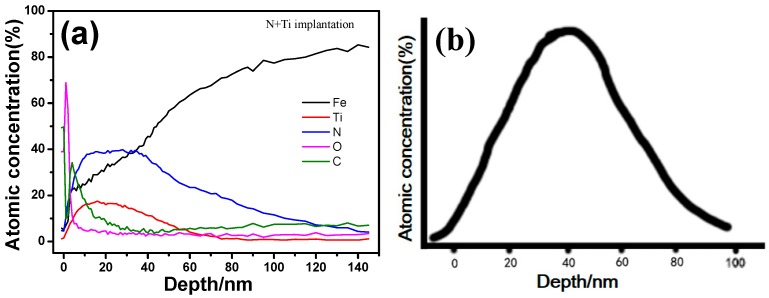
(**a**) The distribution of atomic concentration along the depth of N + Ti implanted sample and (**b**) corresponding stimulation results of Ti implanted in the M50 substrate (parameters for simulation as same as real implantation: Energy: 90 keV, 60 keV and 40 keV at 45 kV, 30 kV and 20 kV of accelerating voltage; implantation dose: 2 × 10^17^ ions/cm^2^).

Atomic concentration distribution along the depth (Figure 6a) shows that the peak concentration of N and Ti is in the range of 5–40 nm. Specifically for the Ti atom, its peak concentration is at a depth of ~20 nm, and almost vanishes at 50 nm. The results basically agree with those obtained by calculation using SRIM software (v2013, SRIM.com, Annapolis, MD, USA). (Figure 6b). In addition, elements O and C are enriched in the surface layers of depth 3 nm and 10 nm, respectively, and their contents decrease rapidly to the bulk concentration with increasing depth. The segregations of O and C atoms near the surface has been reported previously [21]. It occurs not only in the implanted specimens, but also in the pristine sample, and is possibly related to the atomic diffusion during polishing.

**Figure 7:**
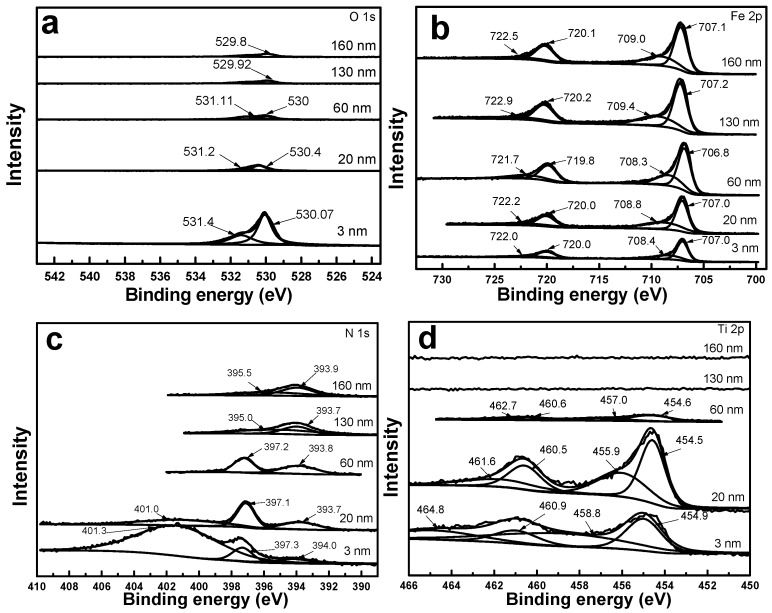
(**a**) O 1s; (**b**) Fe 2p; (**c**) N 1s and (**d**) Ti 2p core energy level spectra at different depths (3 nm, 20 nm, 60 nm, 130 nm, and 160 nm).

Figure 7a shows the O 1s X-ray photoelectron spectra (XPS) at different implantation depths. It indicates that the peak intensity of oxygen was at its maximum at 3 nm, and beyond the thickness of 20 nm, O 1s peak became very weak. At a depth of 3 nm, the two peaks at 530.07 and 531.4 eV corresponded to FeO and TiOx, respectively. The peak due to TiO_2_ disappeared beyond 60 nm, consistent with the depth distribution of the Ti element in the AES results. Oxides were not found in the XRD results, which may be related to the amorphous state.

Figure 7b shows the Fe 2p XPS peak at different depths. Four peaks are observed at a depth of 3 nm, among which the 2p_3/2_ and 2p_1/2_ peaks at 707.0 and 720.0 eV, respectively, are supposed to be the 2p peak of Fe, while the 2p_1/2_ and 2p_3/2_ peaks at 722.0 and 708.4 eV, respectively, correspond to Fe_3_O_4_. In addition, at depths of 20, 60, 130, and 160 nm, the peak intensities of Fe increase gradually with increasing depth, which agrees well with its concentration distribution along the depth. This is because, with increasing depth, oxygen elements that form compounds with Fe gradually decrease in amount and disappear. At depths greater than 60 nm, the peaks at 709.4 eV and 709 eV are attributed to iron carbide [24].

The N 1s core-level spectra (Figure 7c) reveal that TiN was formed in the region of 60 nm, represented by the peak at 397.3 eV. The peak disappears when the Ti element vanishes at depths greater than 60 nm. As reported by [9], the peak at 401 eV can probably be attributed to interstitial N. The N 1s peak intensity of TiN is strongest at 20 nm, consistent with the maximum peak concentration of Ti element in this region. The formation of TiN after implantation of N and Ti has been reported previously [25].

The Ti 2p core-level spectra (Figure 7d) shows peaks at 460.9 eV and 454.9 eV at 3 nm, corresponding to TiN. The peaks at 464.8 and 458.8 eV are attributed to TiO_2_. At 20 nm, the peaks at 460.5 and 454.5 eV correspond to TiN, and those at 461.6 and 455.9 eV are attributed to Ti_2_O_3_. Beyond 60 nm, the characteristic peaks of the Ti element become weak and ultimately disappear, indicating that the Ti element becomes non-existent. Therefore, TiO_x_ and TiN_x_ mainly co-exist in the depth range of 0–20 nm, and the presence of Ti may be mainly due to substitution of Fe [25,26].

The peak concentration of the implanted N element appears at depths of 3–60 nm, and the TiN phase is formed with the Ti element. All these results demonstrate that the compound and microstructure in the implanted layer is in the form of graded distribution.

### 3.4. TEM Microstructure Analysis

**Figure 8:**
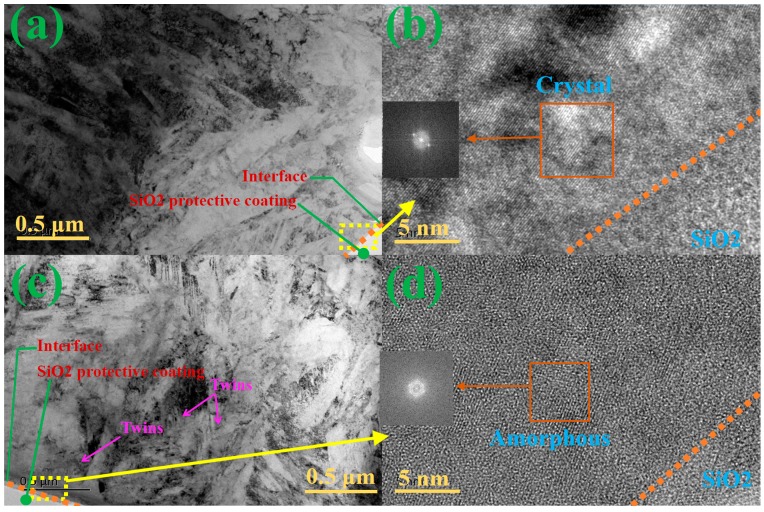
The comparative microstructures of pristine M50 matrix without and with implantation: (**a**) Microstructure with no implantation; (**b**) magnified image showing morphology at transition, corresponding to the yellow box in (**a**); (**c**) Microstructure with implantation; (**d**) magnified image showing morphology at transition, corresponding to the yellow box in (**c**).

The comparative microstructures of the pristine M50 matrix and that with implantation are shown in Figure 8. It is obvious that the lath martensite is the main phase in the M50 steel matrix (Figure 8a). Figure 8b shows the magnified image of the morphology at transition in Figure 8a. The crystal phase and the corresponding diffraction ring are attributed to the lath martensite, and the amorphous SiO_2_ protective coating is used for prevention from oxidation of samples in TEM tests. However, after M50 was implanted with N and Ti, the lath martensite underwent graded refinement, and twins grew beneath the transition layer (Figure 8c). Besides, the refined lath martensite grows bigger along the implantation depth. As shown in Figure 8d, the magnifying special morphologies and their diffraction patterns are illustrated for further investigation. Both amorphous phase and nano-crystal phases can be found in the transition area. The microstructure of the sample below the amorphous layer is nanocrystalline, with the transition layer of a certain thickness (Figure 8c). According to the previous analysis of the depth distribution of the elements, this special transition layer is mainly composed of the TiN ceramic phase and Ti metallic phase, which can cause strengthening by supersaturated solid solution and dislocation tangle. Based on the above investigation, the gradient structure in the transition zone was formed from the top amorphous layer to the refined martensite layer via the middle nanocrystalline layer. Meanwhile, precipitation compounds are also grown by implantation. Consequently, these two effects largely contribute to friction reduction, wear resistance, and nano-hardness.

## 4. Conclusions

The hardness and tribological performance of M50 bearing steels can be improved by ion implantation. In this study, M50 bearing steels were alternately implanted with Ti^+^ and N^+^ ions by solid and gas ion implanters, respectively. The test results showed that the surface implanted by this technology was formed by an amorphous layer at a certain depth below the M50 bearing steel surface, and a modified layer of nanocrystallites and grain-refined crystals was developed beneath the amorphous layer. The distribution of the elements was graded, rather than uniform. The compounds of Ti and TiN phases were formed in the amorphous and nanocrystalline layers. The polyenergy also induces long-range effects to refine lath martensite and form twinning in the sample. Along the implantation depth, the grain also displays gradient refinement, which improves the friction and wear properties and the hardness of the modified layer. The nano-indentation hardness of N + Ti-co-implanted sample is above 12 GPa, which is ~1.3-times that of the pristine samples. The friction coefficient is smaller than 0.2, which is 22.2% of that of the pristine samples. This bi-ion polyenergy implantation method may be a useful strategy for enhancing the mechanical performances not only in bearing components but also in other engineering materials. In future studies, performances of other steels (composed of different elements and having different micro-structures) will be considered.

## Figures and Tables

**Table 1:** Chemical composition of M50 material (mass percentage).

Element	C	Cr	Mo	V	Mn	Si	P	Fe
Content%	0.8	3.97	4.37	1.04	0.1	0.2	0.01	allowance

**Table 2:** Processing parameters of N^+^ and Ti^+^ bi-ion implantation.

Implantation Parameter	Parameter Values
Base vacuum	3.0 × 10^−4^ Pa
Sample temperature	150–180 °C
N ion acceleration voltage	80 kV
N ion implantation dose	2 × 10^17^ ions/cm^2^
Ti ion acceleration voltage	45–20 kV
Ti ion implantation dose	2 × 10^17^ ions/cm^2^
